# Comparison of whole-body computed tomography vs selective radiological imaging on outcomes in major trauma patients: a meta-analysis

**DOI:** 10.1186/s13049-014-0054-2

**Published:** 2014-09-02

**Authors:** Libing Jiang, Yuefeng Ma, Shouyin Jiang, Ligang Ye, Zhongjun Zheng, Yongan Xu, Mao Zhang

**Affiliations:** 1 Department of Emergency Medicine, Second Affiliated Hospital, School of Medicine & Institute of Emergency Medicine, Zhejiang University, No 88, Jiefang Rd, Hangzhou 310009, China

**Keywords:** Whole-body CT, Meta-analysis, Mortality, Trauma

## Abstract

**Introduction:**

The purpose of this meta-analysis was to explore the value of whole-body computed tomography (WBCT) in major trauma patients (MTPs).

**Methods:**

A comprehensive search for articles from Jan 1, 1980 to Dec 31, 2013 was conducted through PubMed, Cochrane Library database, China biology medical literature database, Web of knowledge, ProQuest, EBSCO, OvidSP, and ClinicalTrials.gov. Studies which compared whole-body CT with conventional imaging protocol (X-ray of the pelvis and chest, trans-abdominal sonography, and/or selective CT) in MTPs were eligible. The primary endpoint was all-cause mortality. The second endpoints included: time spent in the emergency department (ED), the duration of mechanical ventilation, ICU and hospital length of stay (LOS), the incidence of Multiple Organ Dysfunction Syndrome (MODS) /Multiple Organ Failure (MOF). Analysis was performed with Review Manager 5.2.10 and Stata 12.0.

**Results:**

Eleven trials enrolling 26371 patients were analyzed. In MTPs, the application of WBCT was associated with lower mortality rate (pooled OR: 0.66, 95% CI: 0.52 to 0.85) and a shorter stay in the ED (weighted mean difference (WMD), −27.58 min; 95% CI, −43.04 to −12.12]. There was no effect of WBCT on the length of ICU stay (WMD, 0.95 days; 95% CI: −0.08 to 1.98) and the length of hospital stay (WMD, 0.56 days; 95% CI: −0.03 to 1.15). Patients in the WBCT group had a longer duration of mechanical ventilation (WMD, 0.96 days, 95% CI: 0.32 to 1.61) and higher incidence of MODS/MOF (OR, 1.44, 95% CI: 1.35-1.54; *P* = 0.00001).

**Conclusions:**

The present meta-analysis suggests that the application of whole-body CT significantly reduces the mortality rate of MTPs and markedly reduces the time spent in the emergency department.

## Introduction

Trauma is the leading cause of death among people aged 1 to 45 years. In 2010, nearly 5.1 million people died from injuries [1]-[3]. Early diagnosis and treatment are usually the key elements to major trauma patients (MTPs) [4]. There have been studies reporting that a reduction of the diagnostic interval is associated with better prognosis [5]-[7]. Regarding this, recent guidelines for the management of bleeding and coagulopathy recommend that the time elapsed between injury and operation should be minimized [8]. Plain X-rays of the chest and pelvis, focused assessment sonograph trauma (FAST), and organ-specific computed tomography (CT) are conventional evaluation methods in the early diagnostic work-up in MTPs which is recommended by the Advanced Trauma Life Support (ATLS®) protocol [9],[10]. However, it often results in misdiagnosis of some potential life-threatening solid organ injuries and is time-consuming [4],[11]-[16].

During the last decades, CT has played a pivotal role in the early evaluation of MTPs. High resolution multi-slice CT (HRCT) is more sensitive in detecting various occult injuries, especially those potentially life-threatening injuries. Also HRCT is more reliable in excluding underlying injuries [11]-[16]. Meanwhile, the introduction of multi-slice helical CT (MSCT) has significantly shortened the scanning time [17]. In some developed nations, an increasing number of trauma centers use whole-body CT (WBCT) (defined as a CT scan including the head, neck, chest, abdomen, pelvis, and spine) as an early evaluation tool in MTPs. Moreover, in some trauma centers, CT scanner has been located within the emergency department or trauma bay so as to reduce the time from patient’s arrival to WBCT [18]. Recently, clinicians have recognized the advantages of WBCT (especially its diagnostic superiority and time gain) [19],[20]. In addition, there are growing studies indicate that the integration of WBCT into the early assessment protocol significantly increases the probability of survival in those who are severely injured [18]-[23]. Huber-Wagner and colleagues reported that WBCT during trauma resuscitation is associated with a lower risk of mortality in patients regardless of their hemodynamic status [18],[19]. Patients with unstable hemodynamics can be left in their single position to complete the WBCT [4].

Although the proportion of use of WBCT in major trauma has been increased (from 5% in 2002 to 46% in 2010) [24],[25], greater radiation expose becomes one of the major concern for the public because it may induce potential adverse outcomes. For example, the risk of dying from radiation induced cancer is estimated to be 0.08% after one single WBCT in a 45-year old patient [26]. And 1.5%-2.0% malignant tumors are associated with radiation expose in CT scan in America [27]. However, in the field of trauma, it remains inconclusive whether WBCT should be used as an initial assessment tool in MTPs. [28],[29]. Two previous published meta-analysis reported that the application of immediate WBCT has no effect on mortality in MTPs [30],[31]. Recently, several large-scale studies have indicated that the integration of WBCT into initial trauma management can decrease mortality in MTPs. As such, it is high time that those findings should be systematically analyzed.

## Methods

The present meta-analysis was performed according to the preferred reporting items for systematic reviews and meta-analysis (PRISMA) statement [32] (Additional file 1).

### Search strategy

A systematical search of literatures was performed until December 2013, using Cochrane Library database, PubMed, Web of knowledge, ProQuest, EBSCO, OvidSP, China Biology Medicine (http://www.Sinomed.ac.cn) and http://www.clinicaltrials.gov. The following keywords and medical subject headings were used: WBCT, FBCT, TBCT, whole body CT, full body CT, total body CT, pan scan, pan CT, whole body computed tomography, MSCT, MDCT, multi-slice spiral CT, multi-detector CT, multi-slice spiral computed tomography, multi-detector computed tomography, trauma, wound*, injur*, multiple trauma, multiple injur*, severe injur*, severe trauma, polytrauma, and major trauma. We also checked the reference lists of existing systematic reviews and other eligible studies to minimize potential publication bias. The detailed search strategies are available in Additional file 2.

### Study selection

We included studies that met the following criteria:

1) Patients: adult blunt MTPs (age > 16 years, injury severity score (ISS) > 16).

2) Intervention: WBCT.

3) Comparisons: studies compared WBCT with conventional diagnostic algorithm (NWBCT, including X-rays of the chest and pelvis and FAST followed by selective CT or no CT).

4) Outcomes: The primary endpoint was all-cause mortality rate. The secondary endpoints included: time spent in the emergency department (ED), the duration of mechanical ventilation, ICU and hospital length of stay (LOS), the incidence of Multiple Organ Dysfunction Syndrome (MODS)/Multiple Organ Failure (MOF).

5) Study design: Both randomized and observational studies.

### Data extraction and quality assessment

Data were independently extracted by two reviewers (LBJ AND LGY), using a data extraction sheet. The following data were extracted: characteristics of studies, characteristics of patients, types of intervention and outcomes. If necessary, we would contact the corresponding authors to obtain additional information.

The methodological quality of all eligible studies was assessed using the Newcastle-Ottawa Scale (NOS). (www.ohri.ca/programs/clinical_epidemiology/oxford.asp).

### Statistical analysis

Odds ratios (OR) with 95% confidence intervals (CI) were calculated for categorical variables and weighted mean differences (WMD) were calculated for continuous variables. If the published reports of clinical trials only reported the median, range and the size of the trial, we used these published parameters to estimate the mean and the variance (or standard deviation) for such trials according to the formulas described in the study by Hozo et al. [33]. Heterogeneity was tested by the Chi^2^ test (P < 0.10 indicated statistically significant heterogeneity) and I^2^ statistic (I^2^ value >50% indicated significant heterogeneity). The random-effect model was used when there was significant heterogeneity (I^2^ value >50%); otherwise the fixed random-effect was used [34]. A 2-sided P value < 0.05 was considered statistically significant. Sensitivity analysis was performed for the primary endpoint (mortality). Publication bias was quantitatively detected by Egger’s test [35]. The absence of publication bias is indicated by P value >0.10 in Egger’s test. All statistical analyses were performed using STATA 12.0 software (SERIAL NO.40120519635) and RevMan 5.2.10 (http://tech.cochrane.org/revman/download).

The quality of evidence in this meta-analysis was assessed using the GRADE Guidelines [36],[37]. The quality of evidence is classified into four levels: high, moderate, low, and very low, according to the robustness of the evidence [36],[37]. This process was performed using GRADE pro 3.6 software (http://www.Gradeworkinggroup.org/toolbox/index.htm).

## Results

### Search results

**Figure 1:**
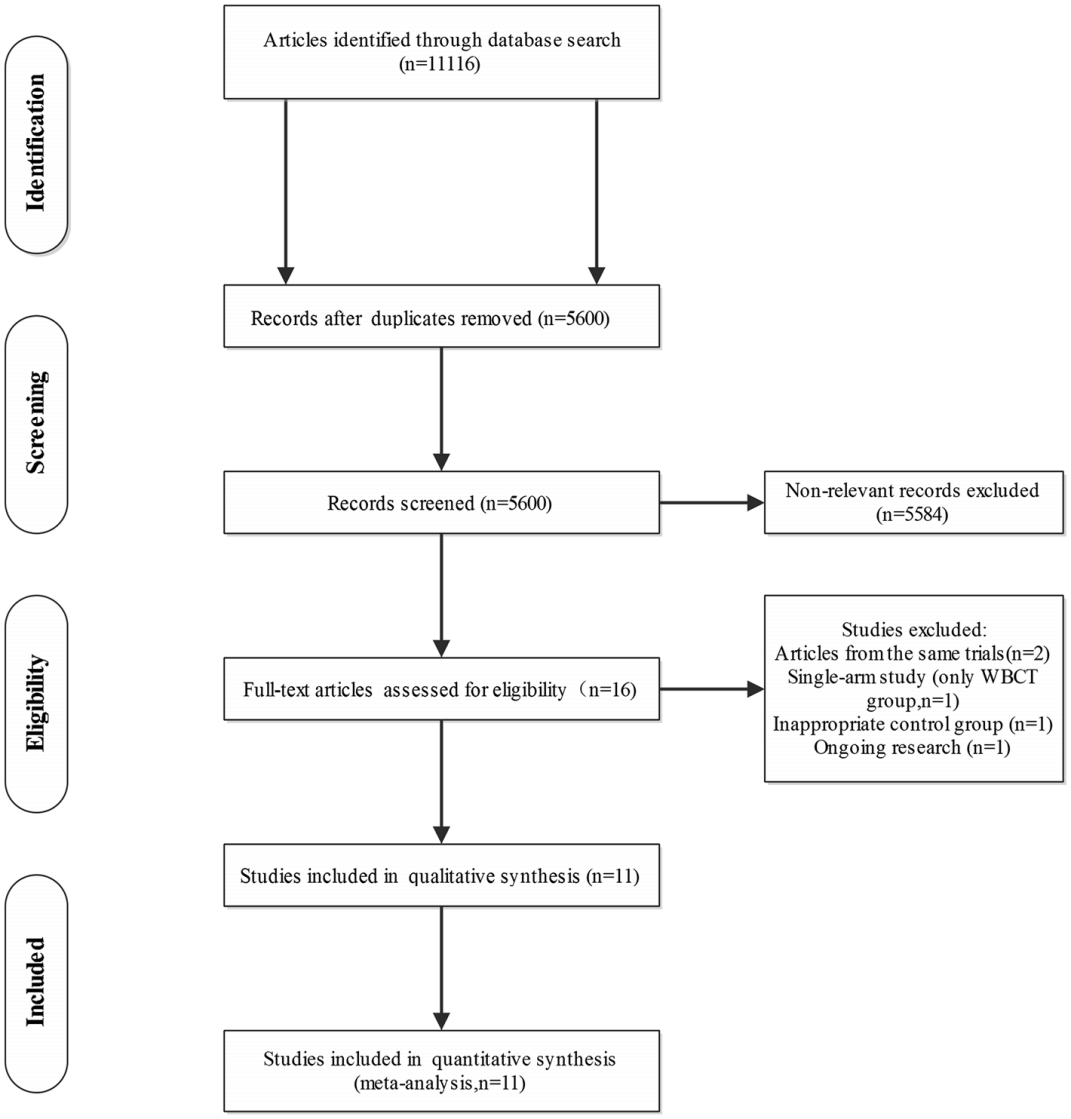
Flow diagram of studies included in this meta-analysis.

We obtained 11116 titles and abstracts through literature search. After screening for abstracts, 11100 duplicated and non-relevant studies were excluded. And the remaining 16 studies were fully read. Of these, 5 studies were excluded and the corresponding reasons were listed in the flow chart (Figure 1). Finally, 11 studies were included in quantitative synthesis [4],[19]-[21],[23],[38]-[43]. Literatures screening process and the reasons for exclusion were shown in Figure 1.

### Study quality assessment

The methodological quality of all included studies was evaluated using the Newcastle-Ottawa Scale. It mainly included the following three aspects: 1) representativeness of the cohort. Ten studies received four stars, indicating that the representativeness of the cohorts was good [4],[19]-[21],[23],[38]-[43]. 2) Comparability. Nine studies received a maximum of two stars [19]-[21],[23],[38]-[41],[43], the remaining studies only received one star due to the reason that the exposed (WBCT) and non-exposed (NWBCT) individuals were not matched in the design and/or confounders were not adjusted for in the analysis [4],[42]. 3) Outcome. Six studies received two stars because of insufficient follow-up time [19]-[21],[39],[40],[43]. Detailed information are summarized in the Additional file 3.

### Study characteristics and meta-analysis

#### Mortality rate

**Figure 2:**
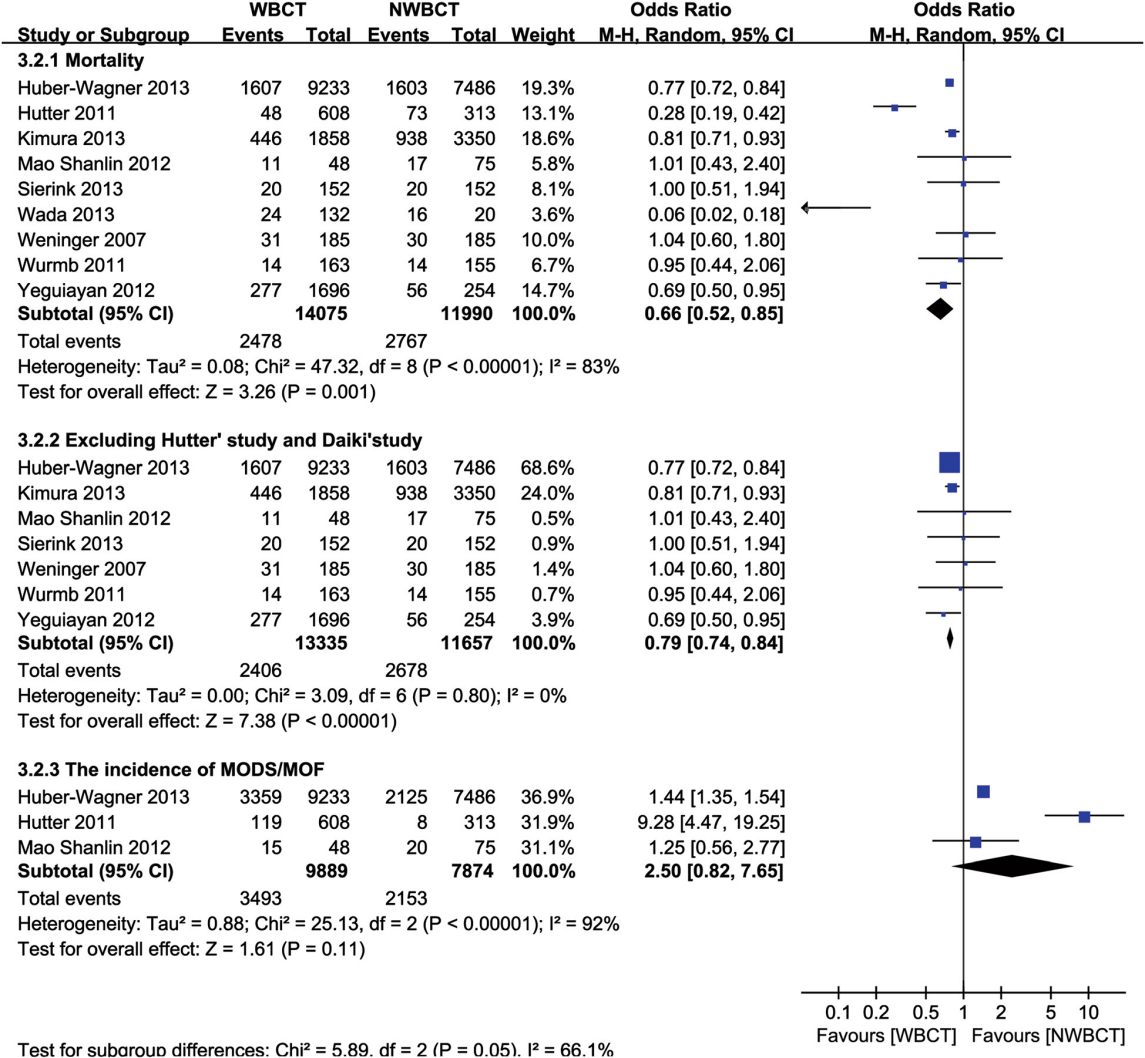
Forest plot for mortality, sensitivity analysis, and the incidence of MODS/MOF.

Ten studies [19]-[21],[23],[38]-[43] reported data on mortality, including four multi-center studies [19],[23],[40],[41] and six single-center studies [20],[21],[38],[39],[42],[43]. The characteristics and results of these studies were summarized in Table 1. Among the included 26210 patients, 14133 patients underwent WBCT. The injury severity was measured using ISS. Patients in the WBCT group had higher ISS than those in the control group in five studies [19]-[21],[40],[41]. On the contrary, in the study by Wada et al. [23], patients in the NWBCT group had higher ISS than those in the WBCT group. In the study of Hutter et al. [21], 831 patients were included after the introduction of a CT-scan policy. However, nearly 27% of these patients did not undergo WBCT, probably because they were less severe than other patients. Thus these patients were excluded so as to avoid selection bias [21],[28]. One of Huber-Wagner’s studies [18] was excluded due to the included patients in this study were analyzed in a more recent published study [19]. The study by Bi et al. [42] was excluded because the baseline characteristics of two groups were not reported. We used a random-effects model for the meta-analysis of mortality due to significant heterogeneity among the studies (*P* < 0.00001; I^2^ = 82%). The combined OR (0.66, 95% CI: 0.52 to 0.85; *P* =0.001, Figure 2) showed significantly lower mortality in patients with WBCT compared to those with NWBCT. The Egger’s test did not show any evidence of publication bias (*P* = 0.349).

**Table 1:** Characteristics of included studies-mortality

		**Male (%)**		**Age (year)**		**Cases(n)**		**ISS**		**Mortality**		***P*** **value**
**Author**	**Design**	**A**	**B**	**A**	**B**	**A**	**B**	**A**	**B**	**A**	**B**	
Sierink et al. [38]	Re	70.4	71.7	43.91 (19.67)^a^	43.63 (18.61)^a^	152	152	18^c^	18^c^	13.2^‡^	13.2^‡^	1.00
Huber-Wagner et al. [19]	Re	73.0	73.2	45.2 (19.8)^a^	44.6 (20.4)^a^	9233	7486	29.7^a^	27.7^a^	17.4^†^	21.4^†^	<0.001
Weninger et al. [39]	Re	72.4	73.5	43.5 (17.2)^a^	40.7 (18.2)^a^	185	185	26.6^a^	27.6^a^	16.8^†^	16.2^†^	n.s.
Wurmb et al. [20]	Re	75	77	38 (3-87)^b^	38 (2-82)^b^	163	155	27^c^	24^c^	8.5^‡^	9.0^‡^	NA
Hutter et al. [21]	Re	75	74	43.9 (19.3)^a^	43.5 (20.7)^a^	608	313	28.3^a^	26.4^a^	7.9^†^	23.3^†^	<0.001
Wada et al. [23]	Re	65	65	40 (26-61)^c^	42 (23-64)^c^	132	20	34^c^	41^c^	18.1^‡^	80^‡^	<0.001
Yeguiayan et al. [41]	Pro	76.1	73.2	NA	NA	1696	254	NA	NA	16.3^‡^	22^‡^	0.02
Kimura et al. [40]	Re	71	70	48 (47-49)^d^	53 (52-53)^d^	1858	3350	26^a^	23^a^	24^†^	28^†^	<0.001
Mao et al. [43]	Re	75	72	49.67 (17.17)^a^	45.24 (15.51)^a^	48	75	28^a^	25^a^	22.9^†^	22.7^†^	0.974
Bi et al. [42]	Re	NA	NA	NA	NA	58	87	NA	NA	20.7^†^	39.1^†^	<0.05

Re, retrospective; Pro, prospective.

ISS, injury severity score.

^†^Mortality during hospital stay; ^‡^30-day or 28-day mortality.

ISS, injury severity score.

A, WBCT, whole-body computed tomography.

B, NWBCT, no CT or only dedicated CT of one or combined body regions was done (non-whole- body CT).

NA, not available. n.s., not significant.

^a^mean (SD); ^b^median (range); ^c^median (IQR); ^d^median (95% CI).

#### Sensitivity analysis

A sensitivity analysis was conducted to explore potential sources of heterogeneity (Additional file 3). In the studies by Hutter et al. and Wada et al. [21],[23], the OR was very different from that of the rest of the studies. It was probably because of the sample size of the control group was too small (132 vs 20) in the study by Wada et al. [23]. And 223 patients in the study by Hutter et al. were excluded due to relatively low injury severity [21]. However, heterogeneity still persisted (data not shown) even when we added these patients to the analysis model [21]. Therefore, a sensitivity analysis was performed after excluding the study by Hutter et al. [21]. Our results showed that the association between WBCT and lower mortality did not change and the pooled OR was 0.77 (95% CI: 0.63 to 0.94; *P* = 0.009) with significant heterogeneity (I^2^ = 69%). Similarly, a sensitivity analysis was performed after excluding the study by Wada et al. [23], which revealed that WBCT was still significantly associated with lower mortality rate (OR, 0.72, 95% CI: 0.59 to 0.89; *P* = 0.002) with significant heterogeneity (I^2^ = 75%). Finally, a further sensitivity analysis was performed after simultaneously excluding these two studies. After pooling the remaining data, the pooled OR was 0.79 (95% CI: 0.74 to 0.84; *P* < 0.00001), which was similar to the above results with non-significant heterogeneity (I^2^ = 0%) (Figure 2).

As shown in Table 1, there were significant differences in baseline characteristics between two groups, especially the ISS values. Thus a sensitivity analysis was conducted to estimate the robustness of our meta-analysis. Studies were only included in this sensitivity analysis scenario if the study authors adjusted for important confounders, such as ISS values. We obtained adjusted OR and its 95% CI from five studies for the pooled results [19],[21],[38],[40],[41]. The pooled OR was 0.54 (95% CI: 0.37 to 0.79; *P* = 0.001) with significant heterogeneity (I^2^ = 89.7%). A sensitivity analysis was also conducted by excluding the study by Hutter et al. [21], in which the adjusted OR was very different from that of the rest of studies. The pooled OR was 0.81 (95% CI: 0.73 to 0.89; *P* = 0.000) with insignificant heterogeneity (I^2^ = 34.7%; *P* = 0.204). Sensitivity analysis was also conducted by adding the study by Bi et al. [42], which had a high risk of bias. The pooled OR was 0.64 (95% CI: 0.50 to 0.82; *P* = 0.0003) which is similar to 0.66 (95% CI: 0.52 to 0.85; *P* =0.001), indicating that this analysis is reliable.

### Secondary outcomes

#### Time spent in the emergency department (ED)

**Figure 3:**
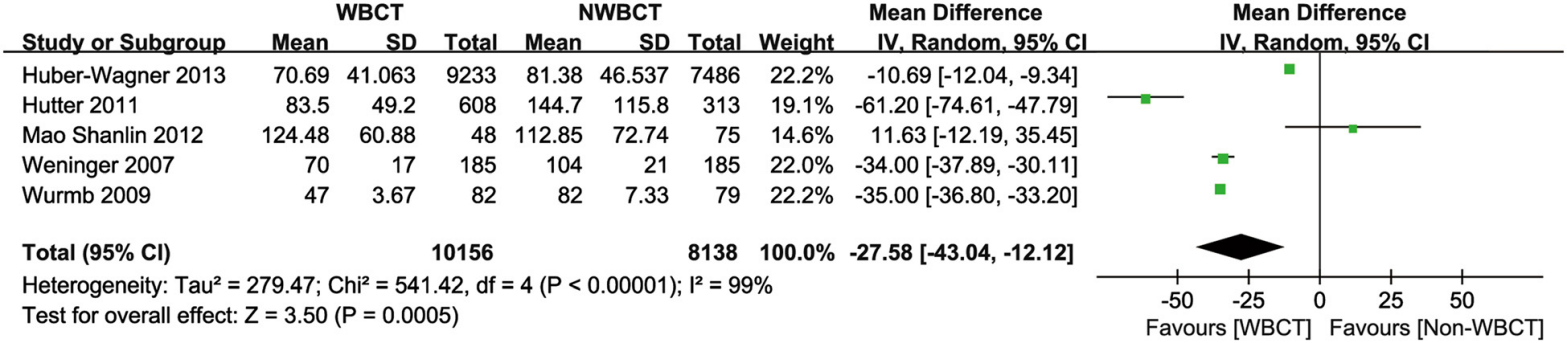
Forest plot for time spent in the emergency department.

Time spent in the ED was reported in six studies (Table 2) [4],[19],[21],[39],[42],[43]. There were no significant differences between the groups in the study by Mao et al. [43], probably because the preparation time for WBCT was too long and the CT room was situated far from the trauma bay. In addition, time spent in the ED was significantly shorter in the WBCT group compared with those in the NWBCT group in the study by Huber-Wagner et al. [19] (Personal communication), Weninger et al. [39], Wurmb et al. [4] and Hutter et al. [21]. The study by Bi et al. [42] was excluded due to insufficient data. Although heterogeneity was present (*P* < 0.00001; I^2^ = 99%), Time spent in the ED was significantly shortened after the introduction of WBCT (WMD = −27.58 min; 95% CI: −43.04 to −12.12; *P* = 0.0005; Figure 3).The Egger’s test demonstrated no evidence of publication bias (*P* = 0.577).

**Table 2:** Characteristics of included studies- ED time

	**Number of cases**		**WBCT ED Time**		**NWBCT ED Time**		***P*** **value**
**Reference**	**WBCT**	**NWBCT**	**Mean/median**	**SD/IQR**	**Mean/median**	**SD/IQR**	
Huber-Wagneret al. [19]	9233	7486	70.69^#^&	41.063^#^&	81.38^#^&	46.53^#^&	<0.05
Weninger et al. [39]	185	185	70^#^	17^#^	104^#^	21^#^	0.025
Wurmb et al. [4]	82	79	47*	37-59*	82*	66-110*	<0.001
Hutter et al. [21]	608	313	83.5^#^	49.2^#^	144.7^#^	115.8^#^	<0.001
Mao et al. [43]	48	75	124.4^#^	60.88^#^	112.8^#^	72.74^#^	0.359
Bi et al. [42]	58	87	32^(NR)^	NR	52^(NR)^	NR	NR

^#^mean/SD; *median/IQR; SD, standard deviation; IQR, interquartile range.

& personal communication.

WBCT, whole-body computed tomography.

NWBCT, no CT or only dedicated CT of one or combined body regions was done (non-whole- body CT).

NR, not reported.

ED, emergency department.

#### ICU -LOS (days)

**Figure 4:**
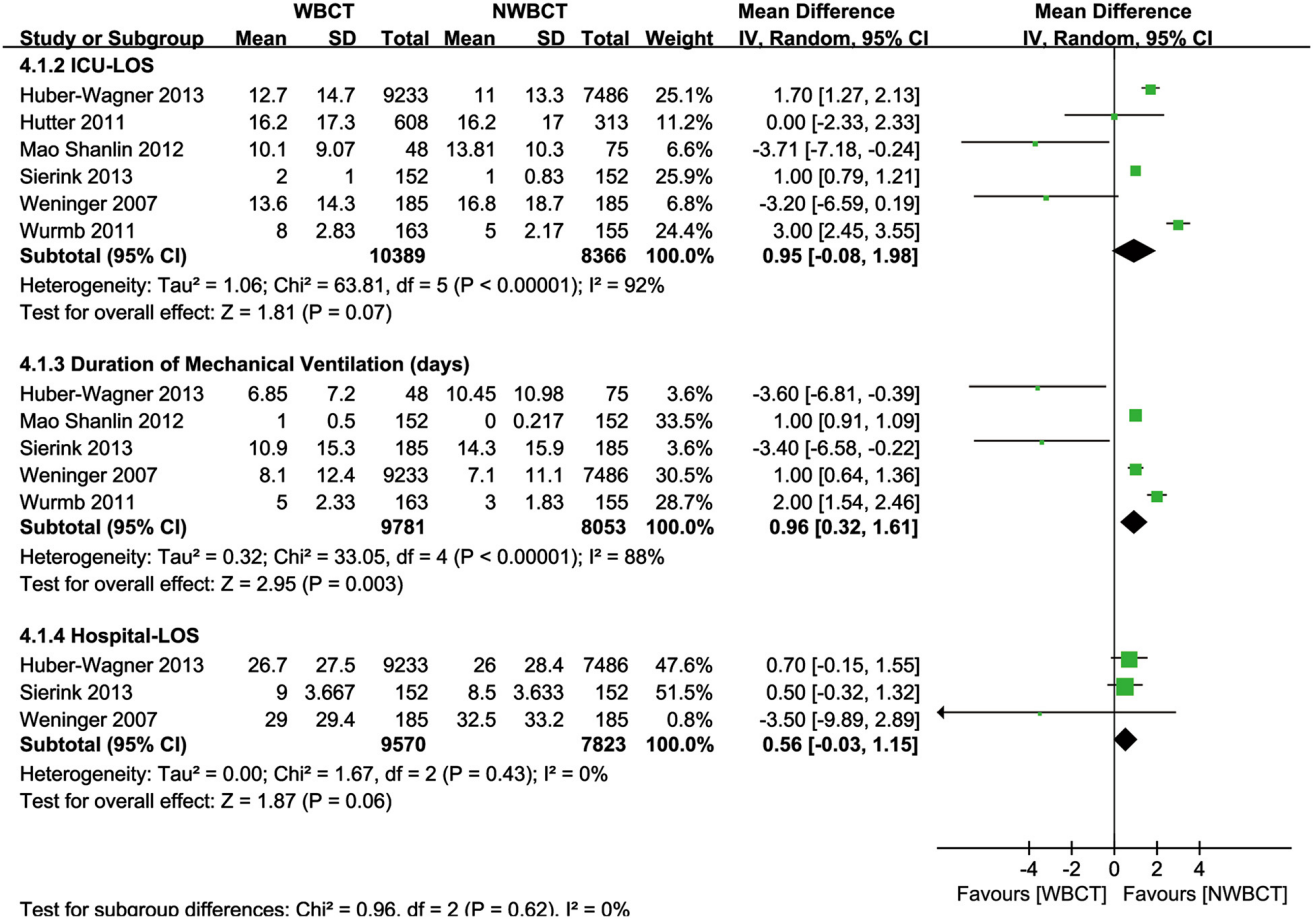
**Forest plot for ICU-LOS, ventilation time, and Hospital-LOS.** LOS, length of stay.

Six studies [19]-[21],[38],[39],[43] reported data on ICU LOS (Table 3). There was no effect of WBCT on the length of ICU stay in the random-effect model (WMD = 0.95 days, 95% CI: -0.08 to 1.98, *P* = 0.07) (Figure 4). There was significant heterogeneity between studies (*P* < 0.00001; I^2^ = 92%). The Egger’s test demonstrated no evidence of publication bias (*P* = 0.855).

**Table 3:** Characteristics of included studies-ICU-LOS/Ventilation time/Hospital-LOS

	**ICU-LOS**			**Ventilation time**			**Hospital-LOS**		
**Author**	**A**	**B**	**P value**	**Author**	**A**	**B**	**P value**	**Author**	**A**
Huber-Wagner et al [19]	12.7 (14.7)*	11 (13.3)*	0.001	8.1 (12.4)*	7.1 (11.1)*	0.001	26.7 (27.5)*	26 (28.4)*	0.001
Sierink et al [38]	2 (0-6)†	1 (0-5)†	0.022	1 (0-3)†	0 (0-13)†	0.134	9 (3-25)†	8.5 (1-22.8)†	0.358
Weninger et al [39]	13.6 (14.3)*	16.8 (18.7)*	0.042	10.9 (15.3)*	14.3 (15.9)*	0.042	29 (29.4)*	32.5 (33.3)*	0.046
Wurmb et al [20]	8 (2-19)†	5 (1-14)†	0.157	5 (1-15)†	3 (1-12)†	0.107	NA	NA	NA
Hutter et al [21]	16.2 (17.3)*	16.2 (17)*	0.05	NA	NA	NA	NA	NA	NA
Mao et al [43]	10.1 (9.07)*	13.81 (10.3)*	0.044	6.85 (7.2)*	10.45 (10.98)*	0.047	NA	NA	NA

†median (IQR ); *mean (SD). SD, standard deviation; IQR, interquartile range.

A, WBCT, whole-body computed tomography.

B, NWBCT, no CT or only dedicated CT of one or combined body regions was done (non-whole- body CT). NA, not available.

#### Duration of mechanical ventilation (days)

Data on the duration of mechanical ventilation were available in five studies [19],[20],[38],[39],[43] (Table 3). The results revealed that the WBCT was associated with longer duration of mechanical ventilation in the random-effect model, the WMD was 0.96 days (95% CI: 0.32 to 1.61, *P* = 0.003) (Figure 4) with significant heterogeneity (*P* < 0.00001; I^2^ = 88%). The Egger’s test demonstrated no evidence of publication bias (*P* = 0.728).

#### Hospital-LOS (days)

Three studies reported data on the length of hospital stay [19],[38],[39] (Table 3). There was no effect of WBCT on the length of hospital stay in the fixed-effect model (WMD, 0.56 days, 95% CI: -0.03 to 1.15; *P* = 0.06) (Figure 4), with no heterogeneity found between studies (*P* = 0.43; I^2^ = 0%). The Egger’s test demonstrated no evidence of publication bias (*P* = 0.185).

#### The incidence of MODS/ MOF

Data on the incidence of MODS/MOF were available in three studies [19],[21],[43] (Table 3). The incidence of MODS/MOF was significantly lower in the NWBCT group compared with the WBCT group in the studies by Huber-Wagner et al. and Hutter et al. [19],[21]. Whereas, there was no significant difference between the two groups in the study by Mao et al. [43]. The incidence of MODS/MOF did not differ between the WBCT group and NWBCT group in the random-effect model (OR, 2.50, 95% CI: 0.82-7.65, *P* = 1.018), with significant heterogeneity found between studies (I^2^ = 92%) (Figure 2). The study by Hutter et al. [21] was excluded because the OR was very different from that of the rest of the studies. The pooled OR suggested that the incidence of MODS/MOF was higher in the WBCT group than in the control group (OR, 1.44, 95% CI: 1.35-1.54; *P* = 0.00001), with no heterogeneity (I^2^ = 0).

### GRADE of evidence

The GRADE of the evidence was summarized in Table 4.

**Table 4:** GRADE evidence profile

	Illustrative comparative risks* (95% CI)			
**Outcomes**	**Assumed risk**	**Corresponding risk**	**Relative effect (95% CI)**	**GRADE**
	NWBCT	WBCT		
Mortality	231 per 1000	190 per 1000	OR 0.66	⊕⊕⊝⊝ low^1,2^
		(180- 200)	(0.52 - 0.85)	
ED Time	The mean ED time ranged across control groups from 82 to 144.7 min	The mean ED time in the intervention groups was 27.58 lower (43.04 to 12.12 lower)	NR	⊕⊕⊝⊝ low^1,2,3,4^
ICU-LOS	The mean ICU-LOS ranged across control groups from 1 to 16.8 days	The mean ICU-LOS in the intervention groups was 0.95 higher (0.08 Lower to 1.98 higher)	NR	⊕⊝⊝⊝ very low^1,2,5^
Ventilation Time	The mean duration of mechanical ventilation ranged across control groups from 0 to 14.3 days	The mean duration of mechanical ventilation in the intervention groups was 0.96 higher (0.32 to 1.61 higher)	NR	⊕⊝⊝⊝ very low^1,2,6^
Hospital-LOS	The mean Hospital- LOS ranged across control groups from 8.5 to 32.5 days	The mean Hospital-LOS in the intervention groups was 0.56 higher (0.03 Lower to 1.15 higher)	NR	⊕⊝⊝⊝ very low^1,2,7^
MODS/MOF	284 per 1000	363 per 1000	OR 1.44	⊕⊝⊝⊝ very low^1,8^
		(348 to 379)	(1.35 to 1.54)	

*The basis for the assumed risk (e.g. the median control group risk across studies) is provided in footnotes. The corresponding risk (and its 95% confidence interval) is based on the assumed risk in the comparison group and the relative effect of the intervention (and its 95% CI). CI: Confidence interval; OR: Odds ratio.

LOS, length of stay; ED time, time spent in the emergency department; NR, not reported.

^1^There were significant differences in baseline characteristics between the two groups (WBCT and NWBCT).

^2^In most of the studies, patients who underwent WBCT had higher levels of injury severity score (ISS).

^3^There was evidence of heterogeneity between studies (Chi^2^ = 541.42, df = 4, P < 0.00001; I^2^ = 99%).

^4^The pooled effect size of weighted mean difference = −27.58 min; 95% CI −43.04 to −12.12; p = 0.0005)

^5^we found a significant heterogeneity among the studies (Chi^2^ = 63.81, df = 5, P < 0.00001; I^2^ = 92%).

^6^The test of heterogeneity showed significant differences in each study (Chi^2^ = 33.05, df = 4, P < 0.00001; I^2^ = 88%).

^7^The amount of studies (total length of hospital stay was described) is too little.

^8^OR was 1.44 [95% CI 1.35-1.54], p < 0.00001.

## Discussion

Our results indicated that the integration of WBCT into the early diagnostic protocol significantly increased the probability of survival in MTPs. We also found that patients in the WBCT group had a significantly shorter ED stay. And there was no effect of WBCT on the length of ICU and hospital stay. To our best knowledge, this is the first meta-analysis of current published trials on the use of WBCT in MTPs.

In addition, patients in the WBCT group have a longer duration of mechanic ventilation and a higher incidence of MODS/MOF, as compared with those in the control group. However, it's not yet clear if there is any true cause-and-effect relationship between the application of WBCT and these adverse outcomes. Firstly, this is probably a result of the reduction of the mortality rate in MTPs. Patients that would have died if without WBCT now survive being aware of the whole injury pattern obtained by WBCT early. Therefore, the higher incidence of MODS/MOF in the WBCT group probably because there are more MTPs survivors in the early phase of hospital care. It has been reported that the classic trimodal death distribution of death following injury (the first peak included immediate deaths, the second peak included early hospital death, and The third peak included the late deaths) has been changed and is much more skewed to early hospital death, largely due to better prehospital and intensive care [44],[45]. Therefore, the biggest challenge is to reduce early hospital mortality. From this perspective, there would seem to be a good rationale for use of WBCT in MTPs to reduce early hospital mortality. Secondly, patients in the WBCT group are more serious (higher ISS values) than those in the NWBCT group which also might account for the higher incidence of MODS/MOF, and longer duration of mechanical ventilation in the WBCT group.

Computed tomography (CT) possesses multiple advantages in MTPs. Firstly, compared with conventional diagnostic approaches, WBCT has higher accuracy especially in the diagnosis of solid organ injuries [39]. Secondly, WBCT can significantly reduce time interval between patient’s arrival and the end of life saving procedures, the end of diagnostic procedures, and the beginning of emergency surgery [4],[20],[39],[46]. A delay of proper surgical care is associated with higher risk of preventable death in trauma care [20]. Rieger et al. reported that it was possible to detect all injuries through WBCT and the time for diagnostic work-up was shortened by 50% as a result of the early use of WBCT. Thirdly, patients in the WBCT group have a shorter ED stay in comparison with those in the control group [4],[39],[46]. Hilbert and colleagues found that a new algorithm that integrating multi-slice CT into the early diagnostic protocol can significantly reduce the length of stay in the trauma room [47].

Some researchers hold that it is reasonable to screen MTPs (ISS ≥16) with WBCT [4],[23],[40],[48],[49]. However, how to accurately identify patients who are severely traumatized remains a problem. The conventional approach is based on injury mechanism, clinical symptom, and physiological indicators [49]. However, under this triage criteria, almost 30% of patients were found to have an ISS below 16 (over-triage) [21],[49]. Unnecessary CT scanning not only can increase the risk of radiation expose, but also is associated with a substantial economic burden [26],[27]. Fortunately with the continuous improvement of CT scanning technology, especially after the introduction of iterative reconstruction techniques, the effective dose of WBCT has decreased from 10-20 mSv to 5-10 mSv [19]. And it has been reported that compared to selective CT, WBCT induces no increased radiation dose in favorable situations [50]. Sierink et al. recently reported that although MTPs (ISS≧16) in the WBCT group experienced higher radiation dose in the trauma room, the total radiation dose throughout hospital admission was comparable between groups [25]. In short, the triage rule may need to be reevaluated.

Some limitations of this study deserve to be mentioned. Firstly, all included studies in this meta-analysis are observational, non-randomized studies. However, based on the available data, and the fact that WBCT has significantly speeded up the diagnosis and treatment process and increased the probability of survival in MTPs, we do not feel that randomized controlled trials (RCTs) can change the current evidence, and it is unethical currently. Secondly, there were significant differences in baseline characteristics between groups, especially the ISS values [19]-[21],[23],[40],[41]. Base-line demographic and clinical characteristics were well matched between the two groups in only two studies [38],[39]. Moreover, in three studies, the number of patients in the NWBCT and WBCT groups varied significantly which would increase the probability of type I error [23],[40],[41]. Thirdly, we cannot attribute the survival benefits to the application of WBCT directly, as it is well known that trauma mortality has been ameliorated in many developed countries due to the improved trauma management (not only the introduction of WBCT). Hutter et al. [21] and colleagues also reported that both the use and the availability of WBCT were associated with a lower risk of all-cause mortality. This indicates that temporal comparison is also a major confounder. Finally, we cannot rule out other residual confounding factors, such as type of the scanners, scanning methods, indications for WBCT, different inclusion criteria, the location of the scanners or potential publication bias.

## Conclusion

Compared with conventional strategies for early diagnosis of major trauma patients, whole-body computed tomography is associated with a decreased mortality rate and can shorten the time spent in the emergency department.

## Abbreviations

MTPs: Major trauma patients

CT: Computed tomography

WBCT: Whole-body computed tomography

ICU-LOS: Intensive care unit length of stay

ED: Emergency department

MODS: Multiple organ dysfunction syndrome

MOF: Multiple organ failure

ICU: Intensive care unit

ISS: Injury severity score

WMD: Weighted mean difference

CI: Confidence interval

OR: Odds ratio

## Competing interests

The authors declare that they have no competing interests.

## Authors’ contributions

Dr Jiang is guarantor of the paper, taking responsibility for the integrity of the work as a whole, from inception to published article. LBJ MZ YFM conceived and designed the experiments. LBJ SYJ LGY performed the literature search and review. LBJ, LGY, MZ, YFM evaluated the quality of literatures. LBJ LGY extracted relevant data. LBJ SYJ MZ YAX conducted the statistical analysis. LBJ SYJ LGY ZZZ wrote the manuscript. LBJ SYJ YFM LGY ZJZ YAX MZ read and approved the final manuscript.

## Additional files

## Supplementary Material

Additional file 1: PRISMA checklists. Click here for file

Additional file 2: Search strategy. Click here for file

Additional file 3: **NOS quality assessment scale and the results of sensitivity analysis of mortality rate.** NOS, Newcastle–Ottawa Quality Assessment Scale. Click here for file

## References

[B1] Heron M Deaths: leading causes for 2008 Natl Vital Stat Rep 2012 60 6 1 94 22827019

[B2] Philipp MO Kubin K Hormann M Metz VM Radiological emergency room management with emphasis on multidetector-row CT Eur J Radiol 2003 48 1 2 4 10.1016/S0720-048X(03)00206-7 14511855

[B3] Lozano R Naghavi M Foreman K Lim S Shibuya K Aboyans V Abraham J Adair T Aggarwal R Ahn SY Alvarado M Anderson HR Anderson LM Andrews KG Atkinson C Baddour LM Barker-Collo S Bartels DH Bell ML Benjamin EJ Bennett D Bhalla K Bikbov B Bin Abdulhak A Birbeck G Blyth F Bolliger I Boufous S Bucello C Burch M Global and regional mortality from 235 causes of death for 20 age groups in 1990 and 2010: a systematic analysis for the Global Burden of Disease Study 2010 Lancet 2012 380 9859 2095 2128 10.1016/S0140-6736(12)61728-0 23245604 PMC10790329

[B4] Wurmb TE Fruhwald P Hopfner W Keil T Kredel M Brederlau J Roewer N Kuhnigk H Whole-body multislice computed tomography as the first line diagnostic tool in patients with multiple injuries: the focus on time J Trauma 2009 66 3 658 665 10.1097/TA.0b013e31817de3f4 19276734

[B5] Hoyt DB Bulger EM Knudson MM Morris J Ierardi R Sugerman HJ Shackford SR Landercasper J Winchell RJ Jurkovich G Death in the operating room: an analysis of a multi-center experience J Trauma 1994 37 3 426 432 10.1097/00005373-199409000-00016 8083904

[B6] Smith W Williams A Agudelo J Shannon M Morgan S Stahel P Moore E Early predictors of mortality in hemodynamically unstable pelvis fractures J Orthop Trauma 2007 21 1 31 37 10.1097/BOT.0b013e31802ea951 17211266

[B7] Martin M Oh J Currier H Tai N Beekley A Eckert M Holcomb J An analysis of in-hospital deaths at a modern combat support hospital J Trauma 2009 66 4 S51 S60 discussion S60-51 10.1097/TA.0b013e31819d86ad 19359971

[B8] Spahn DR Bouillon B Cerny V Coats TJ Duranteau J Fernandez-Mondejar E Filipescu D Hunt BJ Komadina R Nardi G Neugebauer E Ozier Y Riddez L Schultz A Vincent JL Rossaint R Management of bleeding and coagulopathy following major trauma: an updated European guideline Crit Care 2013 17 2 R76 10.1186/cc12685 23601765 PMC4056078

[B9] Postma IL Beenen LF Bijlsma TS Berger FH Heetveld MJ Bloemers FW Goslings JC Radiological work-up after mass casualty incidents: are ATLS guidelines applicable? Eur Radiol 2014 24 3 785 791 10.1007/s00330-013-3072-y 24306424

[B10] Gwinnutt C ATLS approach to trauma management Acta Anaesthesiol Belg 2005 56 4 403 16416957

[B11] Self ML Blake AM Whitley M Nadalo L Dunn E The benefit of routine thoracic, abdominal, and pelvic computed tomography to evaluate trauma patients with closed head injuries Am J Surg 2003 186 6 609 613 discussion 613-604 10.1016/j.amjsurg.2003.08.003 14672766

[B12] Tillou A Gupta M Baraff LJ Schriger DL Hoffman JR Hiatt JR Cryer HM Is the use of pan-computed tomography for blunt trauma justified? A prospective evaluation J Trauma 2009 67 4 779 787 10.1097/TA.0b013e3181b5f2eb 19820586

[B13] Salim A Sangthong B Martin M Brown C Plurad D Demetriades D Whole body imaging in blunt multisystem trauma patients without obvious signs of injury: results of a prospective study Arch Surg 2006 141 5 468 473 discussion 473-465 10.1001/archsurg.141.5.468 16702518

[B14] Watchorn J Miles R Moore N The role of CT angiography in military trauma Clin Radiol 2013 68 1 39 46 10.1016/j.crad.2012.05.013 22824572

[B15] Langner S Fleck S Kirsch M Petrik M Hosten N Whole-body CT trauma imaging with adapted and optimized CT angiography of the craniocervical vessels: do we need an extra screening examination? AJNR Am J Neuroradiol 2008 29 10 1902 1907 10.3174/ajnr.A1261 18784210 PMC8118913

[B16] Atluri S Richard HM 3rd Shanmuganathan K Optimizing multidetector CT for visualization of splenic vascular injury. Validation by splenic arteriography in blunt abdominal trauma patients Emerg Radiol 2011 18 4 307 312 10.1007/s10140-011-0961-8 21614477

[B17] Okamoto K Norio H Kaneko N Sakamoto T Kaji T Okada Y Use of early-phase dynamic spiral computed tomography for the primary screening of multiple trauma Am J Emerg Med 2002 20 6 528 534 10.1053/ajem.2002.34802 12369027

[B18] Huber-Wagner S Lefering R Qvick LM Korner M Kay MV Pfeifer KJ Reiser M Mutschler W Kanz KG Effect of whole-body CT during trauma resuscitation on survival: a retrospective, multicentre study Lancet 2009 373 9673 1455 1461 10.1016/S0140-6736(09)60232-4 19321199

[B19] Huber-Wagner S Biberthaler P Haberle S Wierer M Dobritz M Rummeny E van Griensven M Kanz KG Lefering R Whole-body CT in haemodynamically unstable severely injured patients - a retrospective. multicentre study PLoS One 2013 8 7 e68880 10.1371/journal.pone.0068880 23894365 PMC3722202

[B20] Wurmb TE Quaisser C Balling H Kredel M Muellenbach R Kenn W Roewer N Brederlau J Whole-body multislice computed tomography (MSCT) improves trauma care in patients requiring surgery after multiple trauma Emerg Med J 2011 28 4 300 304 10.1136/emj.2009.082164 20659885

[B21] Hutter M Woltmann A Hierholzer C Gartner C Buhren V Stengel D Association between a single-pass whole-body computed tomography policy and survival after blunt major trauma: a retrospective cohort study Scand J Trauma Resusc Emerg Med 2011 19 73 10.1186/1757-7241-19-73 22152001 PMC3267654

[B22] Kanz KG Paul AO Lefering R Kay MV Kreimeier U Linsenmaier U Mutschler W Huber-Wagner S Trauma management incorporating focused assessment with computed tomography in trauma (FACTT) - potential effect on survival J Trauma Manag Outcomes 2010 4 4 10.1186/1752-2897-4-4 20459713 PMC2880019

[B23] Wada D Nakamori Y Yamakawa K Yoshikawa Y Kiguchi T Ogura H Kuwagata Y Shimazu T Tasaki O Hamasaki T Fujimi S Impact on survival of whole-body computed tomography before emergency bleeding control in patients with severe blunt trauma Crit Care 2013 17 4 R178 10.1186/cc12861 24025196 PMC4057394

[B24] Ruchholtz S Lefering R Paffrath T Oestern HJ Neugebauer E Nast-Kolb D Pape HC Bouillon B Reduction in mortality of severely injured patients in Germany Dtsch Arztebl Int 2008 105 13 225 231 19629200 10.3238/arztebl.2008.0225 PMC2696771

[B25] Sierink JC Saltzherr TP Wirtz MR Streekstra GJ Beenen LF Goslings JC Radiation exposure before and after the introductionof a dedicated total-body CT protocol multitrauma patients Emerg Radiol 2013 20 6 507 512 10.1007/s10140-013-1147-3 23949104

[B26] Loewenhardt B Buhl M Gries A Greim CA Hellinger A Hessmann M Rathjen T Reinert M Manke C Bernhard M Radiation exposure in whole-body computed tomography of multiple trauma patients: bearing devices and patient positioning Injury 2012 43 1 67 72 10.1016/j.injury.2011.10.014 22055141

[B27] Snyder GE Whole-body imaging in blunt multisystem trauma patients who were never examined Ann Emerg Med 2008 52 2 101 103 10.1016/j.annemergmed.2007.03.023 17467119

[B28] Stengel D Frank M Matthes G Schmucker U Seifert J Mutze S Wich M Hanson B Giannoudis PV Ekkernkamp A Primary pan-computed tomography for blunt multiple trauma: can the whole be better than its parts? Injury 2009 40 Suppl 4 S36 S46 10.1016/j.injury.2009.10.035 19895951

[B29] Linsenmaier U Krotz M Hauser H Rock C Rieger J Bohndorf K Pfeifer KJ Reiser M Whole-body computed tomography in polytrauma: techniques and management Eur Radiol 2002 12 7 1728 1740 10.1007/s00330-001-1225-x 12111064

[B30] Sierink JC Saltzherr TP Reitsma JB Van Delden OM Luitse JS Goslings JC Systematic review and meta-analysis of immediate total-body computed tomography compared with selective radiological imaging of injured patients Br J Surg 2012 99 Suppl 1 52 58 10.1002/bjs.7760 22441856

[B31] Healy DA Hegarty A Feeley I Clarke-Moloney M Grace PA Walsh SR Systematic review and meta-analysis of routine total body CT compared with selective CT in trauma patients Emerg Med J 2013 31 2 101 108 10.1136/emermed-2012-201892 23314211

[B32] Moher D Liberati A Tetzlaff J Altman DG Preferred reporting items for systematic reviews and meta-analyses: the PRISMA statement BMJ 2009 339 b2535 10.1136/bmj.b2535 19622551 PMC2714657

[B33] Hozo SP Djulbegovic B Hozo I Estimating the mean and variance from the median, range, and the size of a sample BMC Med Res Methodol 2005 5 13 10.1186/1471-2288-5-13 15840177 PMC1097734

[B34] DerSimonian R Laird N Meta-analysis in clinical trials Contr Clin Trials 1986 7 3 177 188 10.1016/0197-2456(86)90046-2 3802833

[B35] Egger M Davey Smith G Schneider M Minder C Bias in meta-analysis detected by a simple, graphical test BMJ 1997 315 7109 629 634 10.1136/bmj.315.7109.629 9310563 PMC2127453

[B36] Balshem H Helfand M Schunemann HJ Oxman AD Kunz R Brozek J Vist GE Falck-Ytter Y Meerpohl J Norris S Guyatt GH GRADE guidelines: 3. Rating the quality of evidence J Clin Epidemiol 2011 64 4 401 406 10.1016/j.jclinepi.2010.07.015 21208779

[B37] Guyatt GH Oxman AD Schunemann HJ Tugwell P Knottnerus A GRADE guidelines: a new series of articles in the Journal of Clinical Epidemiology J Clin Epidemiol 2011 64 4 380 382 10.1016/j.jclinepi.2010.09.011 21185693

[B38] Sierink JC Saltzherr TP Beenen LF Russchen MJ Luitse JS Dijkgraaf MG Goslings JC A case-matched series of immediate total-body CT scanning versus the standard radiological work-up in trauma patients World J Surg 2013 38 4 795 802 10.1007/s00268-013-2310-4 24170153

[B39] Weninger P Mauritz W Fridrich P Spitaler R Figl M Kern B Hertz H Emergency room management of patients with blunt major trauma: evaluation of the multislice computed tomography protocol exemplified by an urban trauma center J Trauma 2007 62 3 584 591 10.1097/01.ta.0000221797.46249.ee 17414332

[B40] Kimura A Tanaka N Whole-body computed tomography is associated with decreased mortality in blunt trauma patients with moderate-to-severe consciousness disturbance: a multicenter, retrospective study J Trauma Acute Care Surg 2013 75 2 202 206 10.1097/TA.0b013e3182905ef7 23702629

[B41] Yeguiayan JM Yap A Freysz M Garrigue D Jacquot C Martin C Binquet C Riou B Bonithon-Kopp C Impact of whole-body computed tomography on mortality and surgical management of severe blunt trauma Crit Care 2012 16 3 R101 10.1186/cc11375 22687140 PMC3580653

[B42] Zhongfu B The value of emergent spiral CT of multiple body regions in severe mutiple trauma patients Modern Medicine 2011 39 4 459 460

[B43] Mao Shanlin X Xinfa XY Hongfei W Lijun L The value of whole-body CT in severe traffic trauma patients during the early resuscitation phase Chinese J Trauma 2012 28 3 269 271

[B44] Evans JA van Wessem KJ McDougall D Lee KA Lyons T Balogh ZJ Epidemiology of traumatic deaths: comprehensive population-based assessment World J Surg 2010 34 1 158 163 10.1007/s00268-009-0266-1 19882185

[B45] Sauaia A Moore FA Moore EE Moser KS Brennan R Read RA Pons PT Epidemiology of trauma deaths: a reassessment J Trauma 1995 38 2 185 193 10.1097/00005373-199502000-00006 7869433

[B46] Wurmb T Balling H Fruhwald P Keil T Kredel M Meffert R Roewer N Brederlau J Polytrauma management in a period of change: time analysis of new strategies for emergency room treatment Unfallchirurg 2009 112 4 390 399 10.1007/s00113-008-1528-3 19159120

[B47] Hilbert P Zur Nieden K Hofmann GO Hoeller I Koch R Stuttmann R New aspects in the emergency room management of critically injured patients: a multi-slice CT-oriented care algorithm Injury 2007 38 5 552 558 10.1016/j.injury.2006.12.023 17472791

[B48] Rieger M Sparr H Esterhammer R Fink C Bale R Czermak B Jaschke W Modern CT diagnosis of acute thoracic and abdominal trauma Anaesthesist 2002 51 10 835 842 10.1007/s00101-002-0369-7 12395175

[B49] Wurmb TE Fruhwald P Hopfner W Roewer N Brederlau J Whole-body multislice computed tomography as the primary and sole diagnostic tool in patients with blunt trauma: searching for its appropriate indication Am J Emerg Med 2007 25 9 1057 1062 10.1016/j.ajem.2007.03.016 18022502

[B50] Wedegartner U Lorenzen M Nagel HD Weber C Adam G Diagnostic imaging in polytrauma: comparison of radiation exposure from whole-body MSCT and conventional radiography with organ-specific CT RöFo 2004 176 7 1039 1044 15237348 10.1055/s-2004-813216

