# Supplementary material for: Comparison of whole-body computed tomography vs selective radiological imaging on outcomes in major trauma patients: a meta-analysis
Source: Scand J Trauma Resusc Emerg Med. 2014 Sep 2;22:54. doi: 10.1186/s13049-014-0054-2 (PMC4347587; doi:10.1186/s13049-014-0054-2)
Supplement: Additional file 2: — Search strategy. [file s13049-014-0054-2-S2.pdf]

Additional file 1 Search strategy

PUBMED Search Strategy
#31:#29 AND #30
#30: (""1980""[Date - Publication] : ""2013""[Date - Publication])"
#29: #17 AND #28
#28: #18 OR #19 OR #20 OR #21 OR #22 OR #23 OR #24 OR #25 OR #26 OR #27
#27:"Search ""Wounds and Injuries""[Mesh] OR ""Multiple Trauma""[Mesh]"
#26:"Search severe injur*[Title/Abstract]" OR
#25:"Search multiple injur*[Title/Abstract]"
#24:"Search polytrauma[Title/Abstract]"
#23:"Search major trauma[Title/Abstract]"
#22:"Search severe trauma[Title/Abstract]"
#21:"Search multiple trauma[Title/Abstract]"
#20:"Search wound*[Title/Abstract]"
#19:"Search injur*[Title/Abstract]"
#18:"Search trauma[Title/Abstract]"
#17: #1 OR #2 OR #3 OR #4 OR #5 OR #6 OR #7 OR #8 OR #9 OR #10 OR #11 OR #12 OR #13 OR #14 OR #15 OR #16
#16:"Search pan CT[Title/Abstract]"
#15:"Search pan scan[Title/Abstract]"
#14:"Search ""Multidetector Computed Tomography""[Mesh]"
#13:"Search multidetector computed tomography [Title/Abstract]"
#12:"Search multidetector CT [Title/Abstract]"
#11:"Search MDCT [Title/Abstract]"
#10:"Search multislice spiral computed tomography [Title/Abstract]"
#9:"Search multislice spiral CT[Title/Abstract]"
#8:"Search MSCT[Title/Abstract]"
#7:"Search whole body computed tomography[Title/Abstract]"
#6:"Search total body CT[Title/Abstract]"
#5:"Search Full body CT[Title/Abstract]"
#4:"Search whole body CT[Title/Abstract]"
#3:"Search FBCT[Title/Abstract]"
#2:"Search TBCT[Title/Abstract]"
#1:"Search WBCT[Title/Abstract]"

ProQuest Search Strategy
#1: (ab(trauma) OR ab((injury OR injuries)) OR ab((wound OR wounds)) OR ab((multiple trauma OR multiple injuries)) OR ab((severe trauma OR severe injuries)) OR ab((major trauma OR polytrauma)) OR ab((severe injury OR multiple injury)) AND pd(19800101-20131231))
#2: (ab(WBCT) OR ab((FBCT OR TBCT)) OR ab((whole body CT OR full body CT)) OR ab((total body CT OR whole body computed tomography)) OR ab((MSCT OR multislice spiral CT)) OR ab((multislice spiral computed tomography OR MDCT)) OR ab((multidetector computed

tomography OR multidetector CT)) OR ab((pan pan OR pan CT)) AND pd(19800101-20131231))
#3: #1 AND #2

WEB OF KNOWLEDGE Search Strategy

#1:TOPIC: (WBCT) OR TOPIC: (FBCT) OR TOPIC: (TBCT) OR TOPIC: (whole body CT) OR TOPIC: (full body CT) OR TOPIC: (total body CT) OR TOPIC:(whole body computed tomography) OR TOPIC: (MSCT) OR TOPIC:(multislice spiral computed tomography) OR TOPIC: (multislice spiral CT) OR TOPIC: (MDCT) OR TOPIC: (multidetector CT) OR TOPIC: (multidetector computed tomography) OR TOPIC: (pan scan) OR TOPIC: (pan CT)
Timespan=1900-2013
#2:TOPIC: (trauma) OR TOPIC: (injur*) OR TOPIC: (wound*) OR TOPIC: (multiple trauma) OR TOPIC: (multiple injur*) OR TOPIC: (severe trauma) OR TOPIC: (severe injur*) OR TOPIC: (polytrauma) OR TOPIC: (major trauma)
Timespan=1900-2013
#3:#1 AND #2

EBSCO Search Strategy

#1 :AB trauma OR AB injury OR AB injuries OR AB wound OR AB wounds OR AB multiple trauma OR AB severe trauma OR AB major trauma OR AB polytrauma OR AB multiple injury OR AB multiple injuries OR AB (severe injury OR severe injuries)
#2:AB WBCT OR AB TBCT OR AB (FBCT OR pan scan OR pan CT) OR AB whole body CT OR AB full body CT OR AB total body CT OR AB whole body computed tomography OR AB MSCT OR AB multislice spiral CT OR AB (MDCT OR multidetector computed tomography) OR AB multidetector CT OR AB multislice spiral computed tomography
#3:#1 AND #2

OVIDSP

#1:(trauma or injur$3 or wound$1 or multiple trauma or multiple injur$3 or severe trauma or severe injur$3 or major trauma or poly trauma).mp. [mp=tx, bt, bo, ti, ab, ct, mc, st, or, tn, ps, ds, cb, rn, sq, mq, ge, tm, mi, bc, cc, gl, gn, ot, hw]
#2:exp injury/
#3:1 or 2
#4:(WBCT or FBCT or TBCT or whole body CT or whole body computed tomography or full body CT or total body CT or MSCT or MDCT or pan scan or pan CT or multidetector CT or multislice spiral CT or multislice spiral computed tomography or multidetector computed tomography).mp. [mp=tx, bt, bo, ti, ab, ct, mc, st, or, tn, ps, ds, cb, rn, sq, mq, ge, tm, mi, bc, cc, gl, gn, ot, hw]
#5:3 and 4
#6:Limit 5 to 1980-2013
